# Editorial: Molecular pathways and novel therapeutic targets in regenerative dentistry

**DOI:** 10.3389/fdmed.2024.1426500

**Published:** 2024-05-20

**Authors:** Bruno Cavalcanti, Waruna Lakmal Dissanayaka, Yusuke Takahashi

**Affiliations:** ^1^ School of Dentistry, University of Michigan, Ann Arbor, MI, United States; ^2^ Faculty of Dentistry, The University of Hong Kong, Pokfulam, Hong Kong SAR, China; ^3^ Graduate School of Dentistry, Osaka University, Suita, Japan

**Keywords:** dentin, pulp, regenerative dentistry, signaling pathway, molecular pathway

Editorial on the Research Topic Molecular pathways and novel therapeutic targets in regenerative dentistry

In the last two decades, Dentistry has shifted its focus from repairing tissues to regenerating tissues. This can be seen in all areas, from studies looking into the regeneration potential of gingiva, periodontal ligament, and bone to studies focused on dental tissues such as dentin and pulp (1). Most of these changes have come from the advent of stem cells in dental tissues (2), which brought new insights and alternatives to allow for advanced therapeutic options.

To develop these advanced options, it is well established that tissue engineering and regeneration depend on three factors: cells, scaffolds, and extracellular molecules (3). In this context, much has been published on how the stem cells obtained from the dental and surrounding tissues are adequate for the regeneration of these same tissues (4). For example, dental pulp stem cells are very effective in regenerating the pulp tissue and, consequently, dentin in both *in vitro* and *in vivo* conditions (5, 6). In addition to the cell potential, scaffolds have also been deeply studied with many variations regarding their composition and mode of application (solid, 3D printed, hydrogels, etc.), always looking into adequate functionality according to the clinical tissue to be regenerated (7). However, while these advances show a direct connection to the clinical applications, understanding the molecular pathways and targets for regeneration still lag behind, mostly due to the complexity of intracellular mechanisms and the infinite array of substances that can be used for cell differentiation and function.

This collection was presented as an alternative to gathering innovative ways of studying, interpreting, and applying knowledge on molecular pathways to regenerate dental tissues. It is well known that, without proper signaling, cells would not be able to differentiate and perform adequately. An important example is the fact that, while dental pulp stem cells can differentiate into odontoblasts, it is also necessary that these cells also form new blood vessels to allow for the regenerated pulp tissue to have proper nutrition (8). It is crucial to not only regenerate lost cells and tissues but also maintain the functionality of the regenerated tissue. Therefore, it is essential to have an understanding of the critical signaling pathways and utilize molecules that activate these pathways.

This collection brings some additional insights on this critical piece of the regeneration puzzle either in the review form (Zhou et al.) or in the original research form. The review covers six major pathways involved in the odontogenic differentiation of DPSCs: Wnt signaling pathways, Smad signaling pathways, MAPK signaling pathways, NF-kB signaling pathways, PI3K/AKT/mTOR signaling pathways, and Notch signaling pathways. This collection also comprised of a study especially focusing on the sonic hedgehog–patched–gli signaling pathway that covers the stem cell differentiation into pulp tissue (Ishikawa et al.). The bone tissue regeneration area is also covered here, including a study on the application of extracellular substances, such as Resveratrol, to promote bone tissue formation (Hwang et al.). In addition, this collection includes an assessment of single-cell level signaling interactions that guide human odontoblast and ameloblast development to determine incisor or molar tooth germ type identity. In this study, molecular mechanisms were analyzed in the form of induced pluripotent stem cells (iPSC), which also have become an important alternative for tissue regeneration (Hanson-Drury et al.). Finally, the collection brings an important point of view on how to stimulate the new generation of dentists to go deeper into the knowledge of regenerative dentistry and understand how to integrate the progress in the field of regenerative dentistry into the clinical practice guidelines (Jamal and Elhussein).

The papers discussed here signify the tremendous potential of regenerative dentistry in transforming dental treatments. We hope the readers will enjoy this collection and enrich their understanding of molecular aspects and novel therapeutic targets in regenerative dentistry.
